# Supplementary material for: HIVprotI: an integrated web based platform for prediction and design of HIV proteins inhibitors
Source: J Cheminform. 2018 Mar 9;10:12. doi: 10.1186/s13321-018-0266-y (PMC5845081; doi:10.1186/s13321-018-0266-y)
Supplement: Supplementary file 2 — Additional file 2. Source code of HIVProtI web server. [file 13321_2018_266_MOESM2_ESM.zip › HIVprotI_Source-code/hivprotI/weka-3-6-6/doc/weka/clusterers/forOPTICSAndDBScan/OPTICS_GUI/SERObject.html]

SERObject


---


|  |  |  |
| --- | --- | --- |
| **Overview** \| **Package** \| **Class** \| **Tree** \| **Deprecated** \| **Index** \| **Help** \| **Weka's home** | |  |
| **PREV CLASS**   NEXT CLASS | **FRAMES**    **NO FRAMES**     **All Classes** |  |
| SUMMARY: NESTED \| FIELD \| CONSTR \| METHOD | DETAIL: FIELD \| CONSTR \| METHOD |  |


---


## weka.clusterers.forOPTICSAndDBScan.OPTICS\_GUI Class SERObject

```
java.lang.Object
  weka.clusterers.forOPTICSAndDBScan.OPTICS_GUI.SERObject
```

**All Implemented Interfaces:**: java.io.Serializable, RevisionHandler

---

``` public class SERObject extends java.lang.Object implements java.io.Serializable, RevisionHandler ```

SERObject.java

**Version:**
:   $Revision: 1.4 $

**Author:**
:   Zhanna Melnikova-Albrecht (melnikov@cip.ifi.lmu.de), Rainer Holzmann (holzmann@cip.ifi.lmu.de)

**See Also:**: Serialized Form

---

**Constructor Summary**

`SERObject(FastVector resultVector,
int databaseSize,
int numberOfAttributes,
double epsilon,
int minPoints,
boolean opticsOutputs,
java.lang.String database_Type,
java.lang.String database_distanceType,
int numberOfGeneratedClusters,
java.lang.String elapsedTime)`


| **Method Summary** | |
| --- | --- |
| `java.lang.String` | `getDatabase_distanceType()`             Returns the distance-type |
| `java.lang.String` | `getDatabase_Type()`             Returns the type of the used index (database) |
| `int` | `getDatabaseSize()`             Returns the database's size |
| `java.lang.String` | `getElapsedTime()`             Returns the elapsed-time |
| `double` | `getEpsilon()`             Returns the value of epsilon |
| `int` | `getMinPoints()`             Returns the number of minPoints |
| `int` | `getNumberOfAttributes()`             Returns the number of Attributes of the specified database |
| `int` | `getNumberOfGeneratedClusters()`             Returns the number of generated clusters |
| `FastVector` | `getResultVector()`             Returns the resultVector |
| `java.lang.String` | `getRevision()`             Returns the revision string. |
| `boolean` | `isOpticsOutputs()`             Returns the flag for writing actions |

**Methods inherited from class java.lang.Object**

`equals, getClass, hashCode, notify, notifyAll, toString, wait, wait, wait`

**Constructor Detail**

### SERObject

```
public SERObject(FastVector resultVector,
                 int databaseSize,
                 int numberOfAttributes,
                 double epsilon,
                 int minPoints,
                 boolean opticsOutputs,
                 java.lang.String database_Type,
                 java.lang.String database_distanceType,
                 int numberOfGeneratedClusters,
                 java.lang.String elapsedTime)
```


**Method Detail**

### getResultVector

```
public FastVector getResultVector()
```

:   Returns the resultVector

    :   **Returns:**: FastVector resultVector

---


### getDatabaseSize

```
public int getDatabaseSize()
```

:   Returns the database's size

    :   **Returns:**: int databaseSize

---


### getNumberOfAttributes

```
public int getNumberOfAttributes()
```

:   Returns the number of Attributes of the specified database

    :   **Returns:**: int numberOfAttributes

---


### getEpsilon

```
public double getEpsilon()
```

:   Returns the value of epsilon

    :   **Returns:**: double epsilon

---


### getMinPoints

```
public int getMinPoints()
```

:   Returns the number of minPoints

    :   **Returns:**: int minPoints

---


### isOpticsOutputs

```
public boolean isOpticsOutputs()
```

:   Returns the flag for writing actions

    :   **Returns:**: True if the outputs are to write to a file, else false

---


### getDatabase\_Type

```
public java.lang.String getDatabase_Type()
```

:   Returns the type of the used index (database)

    :   **Returns:**: String Index-type

---


### getDatabase\_distanceType

```
public java.lang.String getDatabase_distanceType()
```

:   Returns the distance-type

    :   **Returns:**: String Distance-type

---


### getNumberOfGeneratedClusters

```
public int getNumberOfGeneratedClusters()
```

:   Returns the number of generated clusters

    :   **Returns:**: int numberOfGeneratedClusters

---


### getElapsedTime

```
public java.lang.String getElapsedTime()
```

:   Returns the elapsed-time

    :   **Returns:**: String elapsedTime

---


### getRevision

```
public java.lang.String getRevision()
```

:   Returns the revision string.

    :   **Specified by:**: `getRevision` in interface `RevisionHandler`
    :   **Returns:**: the revision


---


|  |  |  |
| --- | --- | --- |
| **Overview** \| **Package** \| **Class** \| **Tree** \| **Deprecated** \| **Index** \| **Help** \| **Weka's home** | |  |
| **PREV CLASS**   NEXT CLASS | **FRAMES**    **NO FRAMES**     **All Classes** |  |
| SUMMARY: NESTED \| FIELD \| CONSTR \| METHOD | DETAIL: FIELD \| CONSTR \| METHOD |  |


---
